# Effects of Optimal Carbohydrase Mixtures on Nutrient Digestibility and Digestible Energy of Corn- and Wheat-Based Diets in Growing Pigs

**DOI:** 10.3390/ani10101846

**Published:** 2020-10-11

**Authors:** Shunfen Zhang, Ruqing Zhong, Lixiang Gao, Zhengqun Liu, Liang Chen, Hongfu Zhang

**Affiliations:** State Key Laboratory of Animal Nutrition, Institute of Animal Science, Chinese Academy of Agricultural Sciences, Beijing 100193, China; zhangshunfen614@163.com (S.Z.); zhongruqing@caas.cn (R.Z.); glxahu@163.com (L.G.); liuzhengqun2015@163.com (Z.L.)

**Keywords:** carbohydrase, in vitro method, digestibility, pig, corn-soybean meal diet, wheat-soybean meal diet, NSP component

## Abstract

**Simple Summary:**

The addition of carbohydrases was an effective strategy to improve the nutrient availability in diets of pigs. This experiment investigated the effects of two optimal carbohydrase mixtures (OCMs) on nutrient digestibility and digestible energy in growing pigs fed corn-based and wheat-based diets. These OCMs were screened based on the corn-based diet and wheat-based diet, respectively, using an in vitro method developed by our research group in previous studies. The results show that the respective OCM improved the total tract digestibility of most macronutrient and digestible energy for pigs fed both corn-based diet and wheat-based diet. These findings are useful for the rational utilization of carbohydrases in the swine industry.

**Abstract:**

This study aimed to evaluate the effects of optimal carbohydrase mixture (OCM) on macronutrients and amino acid digestibility and the digestible energy (DE) in growing pigs fed the corn-soybean meal-based diet (CSM) and the wheat-soybean meal-based diet (WSM). A total of 36 ileal-cannulated pigs (50.9 ± 4.9 kg initial body weight) were allotted to four dietary treatments randomly, which included CSM and WSM diets, and two diets supplied with corresponding OCM. These OCMs were screened using an in vitro method from our previous study. After the five day adaptation period, fecal samples were collected from d six to seven, and ileal digesta samples were collected on d 8 and 10. Chromic oxide was added as an indigestible marker. The results show that the addition of OCM improved the apparent ileal digestibility (AID) of dry matter (DM), ash, carbohydrate (CHO), neutral detergent fiber, and gross energy (GE) and the apparent total tract digestibility (ATTD) of DM, CHO, and GE in CSM diet (*p* < 0.05), but reduced the apparent hindgut disappearance (AHD) of DM in CSM diet (*p* < 0.05). The ATTD of DM, crude protein (CP), ether extract (EE), ash, and GE and the AHD of DM, CP, EE, ash, CHO, and GE in WSM diet were improved by the OCM addition (*p* < 0.05), whereas the AID of DM, CP, ash, CHO, and GE were decreased (*p* < 0.05). The respective DE contents in CSM and WSM diets were increased from 15.45 to 15.74 MJ/kg and 15.03 to 15.49 MJ/kg under the effects of OCM (*p* < 0.05). Similar to the trend of AID of CP, the OCM addition increased the AID and standardized ileal digestibility (SID) of Ile, Thr, and Cys in CSM diet, but decreased the AID and SID of Ile, Phe, Thr, Val, Ala, Pro, Ser, and Tyr in WSM diet. In conclusion, the OCMs screened by an in vitro method could improve the total tract nutrient digestibility and DE for pigs fed corn-based diet or wheat-based diet but had inconsistent effects on the ileal digestibility of nutrients and energy.

## 1. Introduction

Wheat is widely available together with corn, and the wheat-based diet and corn-based diet are two commonly used commercial swine feed types worldwide [1,2]. However, wheat, corn, and co-products from these grains contain considerable quantities of non-starch polysaccharides (NSP), such as arabinoxylans, which may negatively affect the nutrient utilization of feed in pigs [3]. Usually, the addition of carbohydrases is regarded as an effective strategy to reduce the anti-nutritional effects of NSP and improve the performance of pigs [4]. To maximize this efficacy, multi-carbohydrase mixtures targeted at various NSP components of different feedstuffs were advised in many studies [5,6]. However, due to the great variation of NSP components in cereals and a mass of carbohydrase products in the market, it is usually difficult to select the optimal carbohydrase mixture (OCM) [7].

Compared with the in vivo method, which is regarded as time-consuming, expensive, and labor-intensive, the in vitro method could provide a simple and rapid technique to evaluate the efficiency of exogenous enzymes and screen the combination of the optimal enzymes [8]. In our previous studies, two OCMs for corn-soybean meal-based (CSM) diet and wheat-soybean meal-based (WSM) diet were screened, respectively, using a new in vitro method developed by our research group [9,10]. Under the effects of these OCMs, the in vitro ileal dry matter digestibility (IVDMD) was improved by 3.26% in the CSM diet and 3.48% in the WSM diet [11,12]. However, further in vivo study is still needed to determine if the OCM has similar positive effects on nutrient digestion in pigs.

As previously reported, the responses to exogenous enzymes for corn-based diets were often inconsistent with those of wheat-based diets in pigs [13]. This possibly attributes to the structure and physicochemical characteristics of the NSP, especially the variations of soluble NSP and insoluble NSP contents between wheat-based diets and corn-based diets [14,15]. In addition, the effects of exogenous enzymes originated from various bacteria and fungus may also be inconsistent for pigs fed corn- or wheat-based diets [16]. However, limited research studies have compared the effects of carbohydrase mixtures for pigs fed the corn-based diet with the wheat-based diet.

Therefore, this study was conducted to test the hypothesis that two OCMs screened by one new in vitro method had positive effects on the nutrient and energy digestibility in pigs, and these positive effects were consistent for both the corn-based diet and the wheat-based diet. Thus, the objectives of this experiment were to investigate the effects of two OCMs on macronutrient digestibility, amino acid (AA) ileal digestibility, and digestible energy in growing pigs fed two types of basal diets.

## 2. Materials and Methods

The experimental protocol was reviewed and approved by the Institutional Animal Care and Use Committee of the Institute of Animal Sciences, Chinese Academy of Agricultural Sciences (IAS2019-74).

### 2.1. Diets and Optimal Carbohydrase Mixtures

The chemical compositions of main feed ingredients including corn, soybean meal, wheat, and wheat bran in this experiment were analyzed (Table 1). Two common types of basal diets (CSM diet and WSM diet) for pigs were prepared (Table 2). Two additional test diets were mixed using above basal diets with the corresponding OCM, which was screened using an in vitro method. The calculated metabolizable energy contents of the CSM diet and WSM diet were 13.68 MJ/kg and 12.67 MJ/kg, respectively, which all exceed the maintenance energy requirement for pigs in this study. Vitamins and minerals were included in diets to meet or exceed the nutritional requirement of growing pigs (NRC, 2012) [17]. Chromic oxide was added to all diets as an indigestible marker at 0.4%.

Exogenous carbohydrases, including cellulase (6867 U/g), xylanase (33,290 U/g), *β*-mannanase (49,283 U/g), α-galactosidase (2753 U/g), *β*-glucanase (12,076 U/g), and pectinase (1219 U/g), were used in this study. The respective OCM for each of the two basal diets was composed of above 6 exogenous fiber-degrading enzymes and optimized using a series of two-step in vitro trials, based on the stable gastrointestinal digestion fluid of pigs [11,12]. These trials included the following three parts: (1) determining the dose-response of every single exogenous enzyme supplemental level on the IVDMD of the CSM and WSM diets using the one-way randomized experimental design; (2) screening the optimal combinations of 6 single enzymes for 2 corresponding basal diets using the quadratic orthogonal rotation combination design; (3) investigating the effects of OCMs addition on the IVDMD of basal diets and the improvements of IVDMD were 3.26% in CSM diet and 3.48% in WSM diet, respectively [11,12]. The stable mimetic gastrointestinal digestion fluid preparation procedure used in above in vitro trials was developed by our research group [18]. The exogenous enzyme compositions of two OCMs for respective basal diets were presented in U/kg dry matter feed (Table 3).

### 2.2. Animals and Experimental Design

A total of 36 Duroc × Landrace × Large White barrows (Beijing Breeding Swine Center, Beijing, China) with average initial body weight (BW) of 50.9 ± 4.9 kg were randomly allotted to the above 4 dietary treatments according to a completely randomized design with 9 replications per treatment. All pigs were surgically fitted with a simple T-cannula with an inner diameter of 1.5 cm in distal ileum, according to the procedures described by Stein et al. [19] and recovered from surgery without complications.

All pigs were individually housed in stainless steel crates (1.2 m × 1.5 m) and placed in an environmentally controlled room with a temperature of 23 ± 2 °C. Pigs were weighed and the feed allowance was adjusted at 4% of the initial BW of each pig at the beginning of this experiment. Pigs were offered one of two equal portions of a daily feed allowance at 08:00 h and 16:00 h individually every day. Each pig had ad libitum access to feed and water throughout the experiment.

### 2.3. Samples Collection and Chemical Analyses

The initial 5 days of the 10-day experimental period were allowed for pigs to adapt to the diets. Fecal samples were collected via grab sampling from d 6 to 7. On d 8 and d 10 of this experimental period, Whirl-Pak^®^ bags (NASCO, Fort Atkinson, WI, USA) containing 10 mL of 10% formic acid were attached to the T-cannulas to collect ileal digesta from 08:30 h to 16:30 h. The attached bags were inspected every 30 min and the filled bags were changed. Fecal and ileal digesta samples were stored at −20 °C immediately after collection. At the end of the collection, samples of fecal and ileal digesta from each pig were thawed and mixed evenly. Then, the fecal samples were dried at 65 °C for 72 h, and the ileal digesta samples were freeze-dried. Experimental diets, ingredients, fecal samples, and ileal digesta samples were ground to pass through a 0.5 mm screen before analysis.

Dry matter (DM) [20], crude protein (CP) [21], ether extract (EE; method 996.01; AOAC [22]), and ash (method 942.15; AOAC [22]) of all samples were analyzed. The neutral detergent fiber (NDF) and acid detergent fiber (ADF) contents of samples were analyzed using an Ankom-220 fiber analyzer with filter bag techniques (F57) based on the procedure offered by the instrument company (method 6 and method 7, ANKOM Technology, Fairport, NY, USA) [23]. Total carbohydrate (CHO) content was calculated as the following equation: CHO = DM − (CP + EE + ash) [24]. Starch was determined using the Megazyme Total Starch Assay Procedure (Megazyme International Ireland Ltd., Wicklow, Ireland) based on thermostable α-amylase and amyloglucosidase. The content and composition of soluble non-starch polysaccharides (SNSP) and insoluble non-starch polysaccharides (INSP) were measured by gas chromatography-mass spectrometry (Agilent 7890A-5975C equipped with DB-225 capillary column (30 m × 0.25 mm × 0.25 μm), Agilent J&W Scientific, Santa Clara, CA, USA) according to the method described by Theander et al., [25] with minor modification in our research group. The content and composition of total non-starch polysaccharides (TNSP) were the sums of the INSP and SNSP contents. Gross energy (GE) was analyzed by an adiabatic bomb calorimeter (model 6400; Parr Instrument, Moline, IL, USA). The concentrations of AA in diets and ileal digesta samples were analyzed (method 982.30 E (a, b); AOAC [22]). The method of Fenton and Fenton was used to determine chromic oxide content of diets, ileal digesta, and fecal samples [26].

### 2.4. Calculations and Statistical Analysis

The apparent ileal digestibility (AID) and apparent total tract digestibility (ATTD) of nutrients and GE in the test diets were calculated using the index method [27]. Additionally, the ileal digestible energy (IDE) or digestible energy (DE) values were calculated by multiplying the GE by the observed AID or ATTD of GE of the diet [28]. Apparent hindgut disappearance (AHD) was calculated by subtracting values for AID from values for ATTD [29]. The standardized ileal digestibility (SID) of AA was calculated using the following equation [30]:

SID, % = AID + (basal IAA_end_/AA_diet_) × 100

where AA_diet_ is the amino acid concentration of diet (g/kg of DM); basal IAA_end_ is the basal endogenous losses of AA (g/kg of DM intake), which was measured in another experiment using the same batch of pigs (basal IAA_end_ = Arg 0.14, His 0.11, Ile 0.18, Leu 0.21, Lys 0.09, Met 0.04, Phe 0.28, Thr 0.41, Val 0.51, Ala 0.26, Asp 0.49, Cys 0.14, Glu 0.63, Gly 0.61, Pro 2.47, Ser 0.35, Tyr 0.38 g/kg of DM intake; [31]).

In this study, each pig was regarded as an experimental unit. To test the homogeneity of variance and analyze the outliers, the UNIVARIATE module of SAS 9.1 (SAS Inst. Inc., Cary, NC, USA) was used. Then, data were subjected to ANOVA using the MIXED procedure of SAS. The fixed effect of the diet and the random effect of the pig were included in the main model. Contrast was conducted to determine the effects of the factors of basal diets or the OCM addition. Variability in the data was expressed as the standard error means (SEM) and a probability level of *p* < 0.05 was regarded as statistically significant.

## 3. Results

The SNSP, INSP, and TNSP contents in wheat bran and soybean meal were 2.61%, 27.13%, and 29.74% and 1.89%, 18.29%, and 20.18%, respectively, which were greater than those in wheat (1.80%, 8.02%, and 9.82%) and corn (0.60%, 7.04%, and 7.63%; Table 1). The concentrations of arabinose, xylose, glucose, and arabinoxylans in wheat bran were numerically greater than those in other ingredients, and the soybean meal had the numerically greatest galactose and uronic acid contents. The ratio of SNSP to TNSP in wheat was 0.18 and greater than the other three ingredients, which ranged from 0.08 to 0.09.

The factor of diet had significant effects on the AID, ATTD, and AHD of all macronutrients and energy, IDE content, and DE content (*p* < 0.01) in the present study. The AID of DM, NDF, and GE for pigs fed the CSM diet were similar to those of the WSM diet (Table 4). Pigs fed the CSM diet had greater AID of EE and CHO than those of the WSM diet (*p* < 0.05), whereas the AID of ash and ADF for pigs that consumed the WSM diet were greater than those of the CSM diet (*p* < 0.05). The IDE content in the CSW diet was 13.03 MJ/kg and similar to that in the WSM diet (12.95 MJ/kg). The AID of DM, ash, CHO, NDF, and GE in the CSM diet was improved by the addition of OCM (*p* < 0.05). The OCM addition also increased the AID of NDF and ADF for pigs fed the WSM diet (*p* < 0.05). However, the pigs that consumed the WSM diet with the inclusion of OCM had less AID of DM, CP, ash, CHO, and GE (*p* < 0.05) than the pigs fed the OCM-free WSM diet. The IDE content of the CSM diet was increased from 13.03 MJ/kg to 13.45 MJ/kg under the effect of OCM (*p* < 0.01), whereas the IDE content in WSM was decreased by 9.4% with the OCM addition (*p* < 0.01).

The ATTD of DM, EE, ash, CHO, NDF, ADF, and GE of pigs fed the CSM diet were all greater than those of the WSM diet (*p* < 0.05, Table 5). The DE content of the CSM diet was 15.45 MJ/kg and greater than that of the WSM diet (15.03 MJ/kg). There was a 1.71%, 1.35%, and 1.60% increase for the ATTD of DM, CHO, and GE, respectively, for pigs fed the CSM diet under the effect of OCM (*p* < 0.01). Furthermore, positive effects were discovered in the ATTD of DM, CP, EE, ash, and GE in the WSM diet under the OCM addition (*p* < 0.01). There was a 0.29 MJ/kg and 0.46 MJ/kg increase, respectively, for the DE contents of CSM and WSM diets in pigs under the effects of OCM (*p* < 0.05).

Compared with the WSM diet, the CSM diet had greater AHD of DM, EE, ash, NDF, ADF, and GE (*p* < 0.05, Table 6) and similar AHD of CP and CHO. There was no difference in AHD of CP, EE, ash, CHO, NDF, ADF, and GE for pigs fed the CSM diet with or without the OCM addition except the AHD of DM, which was reduced under the effects of OCM (*p* < 0.05). On the contrary, the AHD of DM, CP, EE, ash, CHO, and GE in the WSM diet were all improved by the OCM supplementation (*p* < 0.05), while the AHD of NDF and ADF were not affected by the OCM addition.

As shown in Table 7 and Table 8, most AA ileal digestibility was affected by the factor of diet (*p* < 0.05), except for the AID and SID of Arg and Met. The pigs fed the CSM diet had greater AID and SID of Leu, Ala, and Asp, but less AID and SID of Lys, Phe, Cys, Glu, Gly, Pro, and Tyr compared to the WSM diet (*p* < 0.05). In addition, the AID of His and Thr of pigs fed the CSM diet were also found greater than those of the WSM diet (*p* < 0.05). There were no differences in AID and SID of most AA for pigs fed CSM diet with or without the OCM addition, except that the AID and SID of Ile, Thr, and Cys were improved by the inclusion of OCM (*p* < 0.05). On the contrary, the negative effects of OCM addition to the WSM diet were discovered in AID and SID of Ile, Phe, Thr, Val, Ala, Pro, Ser, and Tyr (*p* < 0.05).

## 4. Discussion

As reported by Bach Knudsen et al., [14] and Chen et al., [32], the INSP contents of the ingredients used in this study were greater than the SNSP content, and the arabinose, xylose, and glucose were also the main NSP components. The TNSP and INSP contents in corn or wheat were less than values reported by Abelilla and Stein [13], but the soybean meal or wheat bran had greater TNSP and INSP contents in this study than those in Abelilla and Stein’s [13] study. As expected, the wheat bran had the greatest SNSP, INSP, and TNSP contents than those of three other ingredients and the ratio of SNSP to TNSP in wheat was the greatest in this study, which agreed with previous reports [13,14].

Dietary fiber is not only difficult to be degraded by endogenous enzymes but is also reported to encapsulate other nutrients, and then negatively affects nutrient and energy digestibility [32]. Therefore, in the present experiment, the total tract digestibility of most macronutrients and energy was found greater in pigs fed the CSM diet than the WSM diet. These results are in agreement with Jaworski et al., [33] and Zhao et al., [34] who reported an increased level of fiber in the diet decreased the ATTD of nutrients and energy. The AID of ADF in the WSM diet was greater than that in the CSM diet and the AID of NDF also had this similar tendency. This was likely a result of the fact that there were more substrates of ADF and NDF in the wheat-based diet than the corn-based diet [13,16]. The AHD of DM, EE, ash, NDF, ADF, and GE were found to be greater in the CSM diet than those of the WSM diet, which agreed with Abelilla and Stein [13]. This indicated that the dietary fiber components in the CSM diet were more fermentable than those in the WSM diet and could release more nutrients in the hindgut section. The observation that the DE content in the CSM diet (15.45 MJ/kg) was greater than that in the WSM diet (15.03 MJ/kg) was possibly due to the fact that the corn-based diet contained less NDF and ADF compared with the wheat-based diet (NRC, 2012) [15]. Additionally, the greater DE content could have been attributed to the greater EE content and increased ileal and total tract digestibility of EE in the CSM diet [35,36].

In the present experiment, the OCM addition to the CSM diet increased the IDE and AID of most macronutrients except EE and ADF; meanwhile, the ATTD of DM, CHO, and GE was improved by the OCM addition. The DE content in the CSM diet was also increased from 15.45 MJ/kg to 15.74 MJ/kg under the effect of the OCM. This is consistent with most previous studies that reported that exogenous fiber-degrading enzymes had positive effects on nutrient digestibility in pigs fed the corn-soybean meal diet [37,38]. The mechanism might be that the OCM broke the long-chain structure of the polymer, removed the side chain groups, released nutrients in the feed, and thus improved the digestibility of nutrients in the CSM diet [39].

In the present study, it is interesting to find that the in vitro method provided an improvement in ileal digestibility of DM (3.48%), while in vivo data show 7% reduction in AID of DM in WSM diet inclusion of OCM. The reason may be that in vitro methods cannot completely simulate the physiological structure and digestive enzymes of animals, so there are certain differences between in vivo and in vitro results [40]. Therefore, in vitro experiments cannot completely replace animal tests and need to be verified in vivo. These results also suggest that in vitro enzyme screening methods may not be applicable to WSM diets. Additionally, the AID of CP, ash, CHO, and GE in the WSM diet was decreased with the inclusion of OCM. Similar results were also found in some previous studies, which found that the exogenous enzyme addition had negative effects on nutrient and energy digestibility in the foregut section [13,41]. However, on the contrary, more studies reported that the exogenous enzymes supplementation could increase the nutrient and energy digestibility for pigs fed wheat and/or wheat by-products diets [42]. As previously reported, the differences in the fiber component and structural arrangement, and the type and quantity of wheat or wheat by-products used may lead to the inconsistent efficacy of carbohydrases [24,39]. Usually, INSP could increase the digesta passage rate, reduce the endogenous enzymes reaction time, and lead to the decrease in nutrient digestibility [43,44]. On the contrary, SNSP could increase the viscosity of digesta and slow down the digesta passage rate [45]. In the present study, the AID of NDF and ADF were all increased under the effect of OCM addition for pigs fed the WSM diet, which might decrease the digesta viscosity in the foregut section and lead to the speedup of digesta flow [43]. Subsequently, that might lessen the reaction time of nutrients with endogenous digestive enzymes and reduce the nutrient ileal digestion for pigs fed the WSM diet in this study [32,36]. The starch content may also be one of the factors affecting the digestibility. The starch source and amylose/pullulan ratio all affect the digestibility of starch and other nutrients in animals [46]. Starches from different grains also respond differently to enzymes. Yang et al., [47] have reported that in wheat and wheat by-product-based diets, the multi-carbohydrase and phytase complex supplementation did not significantly increase the AID and (or) ATTD of starch. In addition, different sources and dosages of exogenous enzymes applied in diets may also have various efficacies [13,16], which also could induce the decrease in nutrient ileal digestion of the WSM diet in this study. Different from the foregut section digestion, the AHD of DM, CP, EE, ash, CHO, and energy in the WSM diet were all improved by the OCM greatly, which also led to the increase in the ATTD of DM, CP, EE, ash, and GE. The DE content was also increased from 15.03 MJ/kg to 15.49 MJ/kg, which was similar to most previous reports [48]. This might be attributed to the possibility that some undigested nutrients in foregut under the effect of OCM could have transferred to hindgut for fermentation by microorganisms [49].

Usually, there are negative effects of high-level fiber on the AA utilization in pigs, which probably occurs due to the increased endogenous AA loss, and low availability of AA in fiber-rich diets [39]. However, the AID and SID of Lys, Phe, Cys, Glu, Gly, Pro, and Tyr, and the AID of Val were found to be decreased in the CSM diet than the WSM diet, whereas the AID and SID of Leu, Ala, and Asp, and the AID of His and Thr were found greater. The AID of CP for pigs fed the CSM diet was also found to be less than those in the WSM diet. As mentioned above, the ileal digestibility of fiber in the WSM diet was greater than those in the CSM diet, which might release more dietary protein in the foregut. Moreover, the greater SNSP content in the WSM diet might slow down the gastric and intestinal emptying process and give the dietary protein increased exposure time to proteolytic enzymes [50]. Furthermore, there was a greater starch content in the WSM diet compared with the CSM diet, which could affect the utilization of AA [39]. Therefore, most AA and CP digestibility for pigs fed the WSM diet was greater than the CSM diet in the current study, although the WSM diet contained greater fiber.

The carbohydrase addition could increase the fiber decomposition, reduce the endogenous losses, enhance the access to protein for endogenous proteases, and improve the hydrolysis of dietary protein [32,39]. In the current experiment, the OCM addition to the CSM diet increased the AID and SID of Ile, Thr, and Cys, and these results are consistent with previous studies [38]. The low-level of dietary fiber in the CSM diet might be not enough for OCM to cause a significant increase in the ileal AA digestibility in this study [51]. Same with the tendency of AID of CP in the WSM diet, the AID and SID of most AA for pigs that consumed the WSM diet were decreased by the addition of OCM, which agreed with Ji et al., [50], in which the AID and SID of Met, Ala, and Ser of pigs were found to be decreased with the supplementation of exogenous enzymes. This result was extremely different from previous studies that used wheat or wheat by-products as the fiber source [42]. As discussed above, possible reasons for the difference might be due to the various fiber component and structural arrangement, the type and quantity of wheat or wheat by-products used, and sources and dosages of exogenous enzymes applied in diets.

## 5. Conclusions

In conclusion, the optimal carbohydrase mixtures optimized by an in vitro method could improve the total tract nutrient and energy digestibility for pigs fed the corn-based diet and wheat-based diet, but had inconsistent effects on the ileal digestibility of nutrients and energy. Herein, we demonstrate that the inclusion of optimal carbohydrase mixtures was characterized as one viable strategy to increase the nutrients and energy digestibility when a corn-based diet was fed to pigs. Thus, the in vitro method based on optimal carbohydrase mixtures works well for corn-based diet, but it is not convincing for wheat-based diet.

## Figures and Tables

**Table 1:** Analyzed nutrient composition and energy of feed ingredients, as-fed basis.

Item	Corn	Soybean Meal	Wheat	Wheat Bran
Dry matter, %	88.84	89.50	88.26	89.97
Crude protein, %	7.95	39.42	14.76	14.94
Ether extract, %	3.85	1.12	1.68	3.70
Ash, %	1.17	5.35	1.85	5.07
Carbohydrate ^1^, %	66.27	34.21	59.61	57.24
Gross energy, MJ/kg	15.06	15.89	14.61	15.53
INSP ^2^, %				
Rhamnose	-	0.41	-	0.12
Ribose	-	0.36	-	-
Arabinose	1.61	1.89	1.55	5.56
Xylose	2.12	1.36	2.47	9.67
Mannose	0.63	2.71	1.32	2.73
Galactose	0.32	3.71	0.20	0.55
Glucose	1.79	5.26	2.31	7.71
Uronic acids	0.55	2.60	0.17	0.79
Arabinoxylans	3.74	3.25	4.02	15.23
Total INSP	7.04	18.29	8.02	27.13
SNSP ^2^, %				
Rhamnose	-	0.14	-	0.01
Ribose	-	0.05	-	-
Arabinose	0.05	0.04	0.25	0.23
Xylose	0.04	0.04	0.39	0.35
Mannose	0.32	1.02	0.67	1.28
Galactose	0.02	0.18	0.09	0.15
Glucose	0.14	0.18	0.04	0.49
Uronic acids	0.03	0.23	0.04	0.10
Arabinoxylans	0.09	0.08	0.64	0.58
Total SNSP	0.60	1.89	1.80	2.61
TNSP ^2^, %				
Rhamnose	-	0.55	-	0.13
Ribose	-	0.41	-	-
Arabinose	1.66	1.93	1.80	5.79
Xylose	2.16	1.40	2.86	10.02
Mannose	0.95	3.73	1.99	4.00
Galactose	0.35	3.88	0.29	0.69
Glucose	1.93	5.44	2.67	8.21
Uronic acids	0.58	2.84	0.21	0.89
Arabinoxylans	3.82	3.33	4.66	15.82
TNSP	7.63	20.18	9.82	29.74
SNSP/TNSP ^3^	0.08	0.09	0.18	0.09

^1^ Carbohydrate = dry matter – (crude protein + ether extract + ash).^2^ INSP, insoluble non-starch polysaccharides; SNSP, soluble non-starch polysaccharides; TNSP, total non-starch polysaccharides. ^3^ The ratio of SNSP content to TNSP content.

**Table 2:** Ingredients and analyzed nutrient composition of experimental basal diets ^1^.

Item	Diet ^2^	
	CSM	WSM
Ingredients, as-fed basis %		
Corn	73.13	-
Wheat	-	76.15
Soybean meal	23.25	10.23
Wheat bran	-	10.00
Limestone	0.91	0.91
Dicalcium phosphate	1.01	1.01
Vitamin-mineral Premix ^3^	1.00	1.00
Salt	0.30	0.30
Chromic oxide	0.40	0.40
Total	100	100
Calculated metabolizable energy, MJ/kg	13.68	12.67
Analyzed composition, dry matter basis		
Dry matter, %	88.87	89.87
Crude protein, %	17.19	16.46
Ether extract, %	3.52	1.92
Ash, %	4.59	4.41
Carbohydrate ^4^, %	63.61	67.03
Starch, %	44.24	46.40
Neutral detergent fiber, %	10.50	11.94
Acid detergent fiber, %	2.83	3.35
Gross energy, MJ/kg	16.46	16.47
Indispensable AA ^5^, %		
Arg	1.10	0.94
His	0.50	0.39
Ile	0.62	0.53
Leu	1.79	1.15
Lys	0.89	0.99
Met	0.18	0.18
Phe	0.90	0.81
Thr	0.72	0.58
Val	0.87	0.78
Dispensable AA ^5^, %		
Ala	1.04	0.65
Asp	1.67	1.15
Cys	0.26	0.28
Glu	3.28	4.04
Gly	0.75	0.71
Pro	1.20	1.37
Ser	0.94	0.80
Tyr	0.68	0.66

^1^ Two additional test diets were mixed using above 2 basal diets with corresponding optimal carbohydrase mixtures, respectively. ^2^ CSM, corn-soybean meal-based diet; WSM, wheat-soybean meal-based diet. ^3^ Provided the following quantities per kilogram of diet: vitamin A, 8250 IU; vitamin D_3_, 825 IU; vitamin E, 40 IU; menadione, 4 mg; biotin, 0.2 mg; choline chloride, 600 mg; folic acid, 2 mg; niacin, 35 mg; _D_-pantothenate, 15 mg; riboflavin, 5.0 mg; thiamin, 1.0 mg; pyridoxine, 2.0 mg; cobalamine, 25 μg; Cu, 50.0 mg as copper sulfate; I, 0.5 mg as potassium iodide; Fe, 80 mg as iron sulfate; Mn, 25.0 mg manganous oxide; Se, 0.15 mg as sodium selenite; and Zn, 100.0 mg as zinc sulfate. ^4^ Carbohydrate = dry matter − (crude protein + ether extract + ash). ^5^ AA, amino acid.

**Table 3:** The exogenous enzymes composition of optimal carbohydrase mixtures for corresponding basal diets ^1^, U/kg of dry matter feed.

Item ^3^	Basal Diet	
	CSM ^2^	WSM ^2^
Cellulase	533.6	1117.9
Xylanase	9983.7	35,087.7
β-mannanase	4080.6	1917.1
α-galactosidase	251.6	305.0
β-glucanase	1014.4	806.7
Pectinase	107.3	133.7

^1^ The data come from our previous in vitro studies and the improvements of in vitro ileal dry matter digestibility were 3.26% in CSM diet and 3.48% in WSM diet, respectively [11,12]. ^2^ CSM, corn-soybean meal-based diet; WSM, wheat-soybean meal-based diet. ^3^ The measured enzymes activities: cellulase 6867 U/g; xylanase 33,290 U/g; *β*-mannanase 49,283 U/g; α-galactosidase 2753 U/g; *β*-glucanase 12,076 U/g; pectinase 1219 U/g.

**Table 4:** Effects of the optimal carbohydrase mixtures on apparent ileal digestibility of nutrients and energy in growing pigs fed two types of diets, %.

Item	Basal Diet		OCM Addition Diet		SEM	*p*-Value	Contrast		
	CSM ^1^	WSM ^1^	CSME ^1^	WSME ^1^			CSM vs. WSM	CSM vs. CSME	WSM vs. WSME
Dry matter	66.63	67.25	69.83	60.23	0.63	<0.001	0.176	<0.001	<0.001
Crude protein	71.16	75.42	73.71	69.99	0.64	<0.001	0.008	0.109	<0.001
Ether extract	58.63	53.31	61.50	51.16	0.96	<0.001	0.007	0.141	0.254
Ash	8.89	13.79	14.54	−7.79	1.76	<0.001	0.040	0.023	<0.001
Carbohydrate	73.52	71.96	76.37	65.53	0.74	<0.001	0.043	<0.001	<0.001
NDF ^2^	45.36	48.72	52.02	56.64	0.93	<0.001	0.056	<0.001	<0.001
ADF ^2^	22.06	28.74	27.21	35.89	1.24	<0.001	0.016	0.066	0.011
Gross energy	70.33	70.58	72.66	63.95	0.60	<0.001	0.667	<0.001	<0.001
IDE ^2^, MJ/kg	13.03	12.95	13.45	11.73	0.12	<0.001	0.445	<0.001	<0.001

^1^ CSM, corn-soybean meal diet; WSM, wheat-soybean meal diet; CSME, corn-soybean meal diet with the inclusion of optimal carbohydrase mixtures; WSME, wheat-soybean meal diet with the inclusion of optimal carbohydrase mixtures. ^2^ NDF, neutral detergent fiber; ADF, acid detergent fiber; IDE, ileal digestible energy.

**Table 5:** Effects of the optimal carbohydrase mixtures on apparent total tract digestibility of nutrients and energy in growing pigs fed two types of diets, %.

Item	Basal Diet		OCM Addition Diet		SEM	*p*-Value	Contrast		
	CSM ^1^	WSM ^1^	CSME ^1^	WSME ^1^			CSM vs. WSM	CSM vs. CSME	WSM vs. WSME
Dry matter	83.19	81.77	84.90	83.92	0.28	<0.001	0.020	0.007	0.001
Crude protein	81.00	81.96	83.08	85.83	0.48	<0.001	0.380	0.069	0.001
Ether extract	62.49	44.03	63.09	54.89	1.47	<0.001	<0.001	0.684	<0.001
Ash	46.68	40.10	48.49	45.93	0.78	<0.001	<0.001	0.271	0.001
Carbohydrate	89.39	87.21	90.74	88.22	0.29	<0.001	<0.001	0.016	0.055
NDF ^2^	59.70	51.63	63.91	54.22	1.12	<0.001	0.001	0.067	0.238
ADF ^2^	56.63	33.67	61.99	31.42	2.70	<0.001	<0.001	0.167	0.544
Gross energy	83.39	82.09	84.99	84.53	0.29	<0.001	0.039	0.015	<0.001
DE ^2^, MJ/kg	15.45	15.03	15.74	15.49	0.06	<0.001	0.001	0.015	<0.001

^1^ CSM, corn-soybean meal diet; WSM, wheat-soybean meal diet; CSME, corn-soybean meal diet with the inclusion of optimal carbohydrase mixtures; WSME, wheat-soybean meal diet with the inclusion of optimal carbohydrase mixtures. ^2^ NDF, neutral detergent fiber; ADF, acid detergent fiber; DE, digestible energy.

**Table 6:** Effects of the optimal carbohydrase mixtures on apparent hindgut disappearance of nutrients and energy in growing pigs fed two types of diets, %.

Item	Basal Diet		OCM Addition Diet		SEM	*p*-Value	Contrast		
	CSM ^1^	WSM ^1^	CSME ^1^	WSME ^1^			CSM vs. WSM	CSM vs. CSME	WSM vs. WSME
Dry matter	16.56	14.52	15.07	23.70	0.69	<0.001	0.006	0.042	<0.001
Crude protein	9.84	6.54	9.38	15.84	0.83	<0.001	0.059	0.789	<0.001
Ether extract	3.86	−9.28	1.59	3.73	1.28	<0.001	<0.001	0.366	<0.001
Ash	37.80	26.31	33.94	53.72	1.99	<0.001	<0.001	0.166	<0.001
Carbohydrate	15.87	15.25	14.37	22.69	0.65	<0.001	0.483	0.104	<0.001
NDF ^2^	14.34	2.90	11.89	−2.42	1.52	<0.001	<0.001	0.401	0.067
ADF ^2^	34.57	4.93	34.78	−4.48	3.51	<0.001	<0.001	0.968	0.069
Gross energy	13.06	11.52	12.33	20.58	0.67	<0.001	0.022	0.279	<0.001

^1^ CSM, corn-soybean meal diet; WSM, wheat-soybean meal diet; CSME, corn-soybean meal diet with the inclusion of optimal carbohydrase mixtures; WSME, wheat-soybean meal diet with the inclusion of optimal carbohydrases mixtures. ^2^ NDF, neutral detergent fiber; ADF, acid detergent fiber; DE, digestible energy.

**Table 7:** Effects of the optimal carbohydrase mixtures on apparent ileal digestibility of amino acids in growing pigs fed two types of diets, %.

Item	Basal Diet		OCM Addition Diet		SEM	*p*-Value	Contrast		
	CSM ^1^	WSM ^1^	CSME ^1^	WSME ^1^			CSM vs. WSM	CSM vs. CSME	WSM vs. WSME
Indispensable AA ^2^									
Arg	91.54	90.03	91.99	89.55	0.37	0.056	0.130	0.652	0.622
His	87.81	86.27	88.33	84.90	0.35	<0.001	0.050	0.514	0.081
Ile	78.17	78.41	79.45	75.93	0.30	<0.001	0.681	0.045	<0.001
Leu	84.44	81.51	84.94	81.56	0.35	<0.001	<0.001	0.434	0.936
Lys	84.64	87.45	85.59	87.94	0.36	<0.001	0.002	0.258	0.543
Met	83.77	83.39	84.91	82.56	0.32	0.066	0.644	0.190	0.323
Phe	83.96	85.28	85.01	83.37	0.22	0.003	0.016	0.057	0.001
Thr	75.04	73.84	77.11	70.11	0.47	<0.001	0.023	<0.001	<0.001
Val	75.20	76.09	76.54	70.88	0.53	<0.001	0.399	0.220	<0.001
Dispensable AA ^2^									
Ala	73.26	60.81	71.87	58.92	1.15	<0.001	<0.001	0.060	0.010
Asp	80.12	74.74	81.04	73.88	0.58	<0.001	<0.001	0.053	0.061
Cys	73.25	80.51	76.71	80.35	0.66	<0.001	<0.001	0.008	0.893
Glu	84.85	89.93	86.45	89.95	0.47	<0.001	<0.001	0.057	0.987
Gly	66.44	68.69	67.65	70.02	0.41	0.011	0.032	0.247	0.193
Pro	75.79	84.56	75.54	78.25	0.75	<0.001	<0.001	0.813	<0.001
Ser	82.20	81.24	82.18	78.96	0.31	<0.001	0.129	0.985	0.001
Tyr	84.32	87.85	84.64	85.15	0.37	<0.001	<0.001	0.697	0.002

^1^ CSM, corn-soybean meal diet; WSM, wheat-soybean meal diet; CSME, corn-soybean meal diet with the inclusion of optimal carbohydrase mixtures; WSME, wheat-soybean meal diet with the inclusion of optimal carbohydrase mixtures. ^2^ AA, amino acids.

**Table 8:** Effects of the optimal carbohydrase mixtures on standard ileal digestibility of amino acids in growing pigs fed two types of diets, %.

Item	Basal Diet		OCM Addition Diet		SEM	*p*-Value	Contrast		
	CSM ^1^	WSM ^1^	CSME ^1^	WSME ^1^			CSM vs. WSM	CSM vs. CSME	WSM vs. WSME
Indispensable AA ^2^									
Arg	92.66	91.37	93.12	90.89	0.37	0.099	0.190	0.652	0.622
His	89.75	88.78	90.26	87.41	0.32	0.005	0.210	0.514	0.081
Ile	80.73	81.46	82.02	78.97	0.29	<0.001	0.232	0.045	<0.001
Leu	85.48	83.14	85.98	83.19	0.31	<0.001	0.001	0.434	0.936
Lys	85.54	88.27	86.49	88.76	0.36	0.001	0.002	0.258	0.543
Met	85.75	85.44	86.89	84.61	0.32	0.079	0.703	0.190	0.323
Phe	86.70	88.38	87.75	86.47	0.22	0.003	0.003	0.057	0.001
Thr	80.08	80.23	82.16	76.50	0.39	<0.001	0.771	<0.001	<0.001
Val	80.41	81.93	81.75	76.73	0.51	<0.001	0.153	0.220	<0.001
Dispensable AA ^2^									
Ala	75.48	64.38	74.09	62.49	1.04	<0.001	<0.001	0.060	0.010
Asp	82.72	78.56	83.64	77.70	0.48	<0.001	<0.001	0.053	0.061
Cys	78.00	85.06	81.46	84.90	0.65	<0.001	<0.001	0.008	0.893
Glu	86.55	91.33	88.15	91.34	0.45	<0.001	<0.001	0.057	0.987
Gly	73.64	76.43	74.86	77.77	0.44	0.002	0.009	0.246	0.193
Pro	94.01	100.68	93.76	94.37	0.63	<0.001	<0.001	0.813	<0.001
Ser	85.48	85.17	85.47	82.89	0.28	<0.001	0.610	0.985	0.001
Tyr	89.30	93.03	89.61	90.33	0.38	<0.001	<0.001	0.697	0.002

^1^ CSM, corn-soybean meal diet; WSM, wheat-soybean meal diet; CSME, corn-soybean meal diet with the inclusion of optimal carbohydrase mixtures; WSME, wheat-soybean meal diet with the inclusion of optimal carbohydrase mixtures. ^2^ AA, amino acids.
